# Detection of Hantaviruses and Arenaviruzses in three-toed jerboas from the Inner Mongolia Autonomous Region, China

**DOI:** 10.1038/s41426-018-0036-y

**Published:** 2018-03-21

**Authors:** Zhiqiang Wu, Jiang Du, Liang Lu, Li Yang, Jie Dong, Lilian Sun, Yafang Zhu, Qiyong Liu, Qi Jin

**Affiliations:** 1 MOH Key Laboratory of Systems Biology of Pathogens, Institute of Pathogen Biology, Chinese Academy of Medical Sciences & Peking Union Medical College, Beijing, China; 2 Collaborative Innovation Center for Diagnosis and Treatment of Infectious Diseases, Hangzhou, China; 3 State Key Laboratory for Infectious Diseases Prevention and Control, National Institute for Communicable Disease Control and Prevention, Chinese Center for Disease Control and Prevention, Beijing, China

Hantaviruses (HVs) and arenaviruses (AreVs) of rodent origin are important causative agents of human diseases. Many types of rodent HVs (e.g., Hantaan virus, HTNV; Seoul virus, SEOV; and Puumala viruses, PUUV) have been confirmed to be the causative agents of hemorrhagic fever with renal syndrome (HFRS) in humans^1,2^. HFRS primarily caused by HTNV and SEOV remains a serious public health problem in China^3,4^. Many AreVs of rodent origin are confirmed to be zoonotic and to cause severe human hemorrhagic fever and related diseases (e.g., Lassa virus, LASV; Machupo virus, MACV; and Lujo virus, LUJV)^5–8^. Novel AreVs recently identified in China are suspected to have zoonotic potential^9,10^.

Rodent-borne HVs of the genus *Orthohantavirus* under the family *Hantaviridae* are divided into three evolutionary clades under two phylogroups that are strictly associated with the subfamilies of their hosts: *Murinae*-related phylogroup III HVs (HTNV, SEOV, etc.) and *Sigmodontinae*- and *Arvicolinae*-related phylogroup IV HVs (Sin Nombre virus, Andes virus, PUUV, Tula virus, etc.)^2^. Similarly, rodent-origin AreVs of the genus *Mammarenavirus* in the family *Arenaviridae* can be divided into the following two complexes: the Old-World complex and the New-World complex. All reported AreVs of the Old-World complex are *Murinae* borne^6^. In China, rodent species in the subfamily *Murinae*, including *Apodemus* species in rural areas (e.g., striped field mice, *A. agrarius*) and *Rattus* species in cities (e.g., Norway rats, *R. norvegicus*), are the main hosts used for monitoring in the national HFRS surveillance network^3^.

In this study, we collected anal swab samples from 59 rodents of the family *Dipodidae* (26 three-toed jerboas, *D. sagitta*; 5 long-eared jerboas, *Euchoreutes naso*, and 28 five-toed jerboas, *Allactaga sibirica*) in Alashan Left Banner, Inner Mongolia Autonomous Region, in May 2014. Samples from the same species were pooled and then used for next-generation sequencing-based virome analysis using HiSeq2500 after processing by viral particle-protected nucleic acid purification and sequence-independent PCR as previously described^11^. All the raw reads of 100 bp in length generated by HiSeq2500 were aligned to the NCBI nonredundant protein database (NR) using BLASTx after filtering the reads by applying previously described criteria^11^. The taxonomy of these aligned reads was parsed using Megan 4—MetaGenome Analyzer (MEGAN4). Simultaneously, different degenerate primers or specific primers targeting L genes (pan-HV primers and pan-Old-World-AreV primers, separately) were used to study the presence and prevalence of these viruses in individual samples^12,13^.

Based on the NR alignment results, in samples from three-toed jerboas, we identified 26 sequence reads that were classified into the family *Hantaviridae*, covered ~22% of the genome sequence, and shared a high percentages of amino acid (aa) identities (93–97%) with known HTNV. We also identified 14,425 sequence reads that were classified into the genus *Mammarenavirus*, covered ~71% of the genome sequence, but shared low percentages of aa identities (35–56%) with all known AreVs. The approximate locations of these reads and the relative distances between the reads were determined based on the alignment results exported with MEGAN 4. The located reads were then used for reads-based PCR to identify the genomes of these viruses. Finally, partial or complete genome sequences of these viruses were obtained (submitted to GenBank as KY370084, MG525544-MG525546, MF642352, and MF642353 for HVs and KY432892, KY432893, and MF642354-MF642356 for AreVs). Pan-HV and pan-Old-World-AreV screening of all samples revealed that two three-toed jerboa samples were HV positive (7.7%) and three three-toed jerboa samples were AreV positive (11.5%). In August 2015, we conducted another sampling in the same location, and anal swab samples and tissue samples from 24 three-toed jerboas, 7 long-eared jerboas, and 15 five-toed jerboas were collected and applied to pan-HV and pan-Old-World-AreV screening. One of the three-toed jerboas was HV positive (4.2%) and one was AreV positive (4.2%) (Supplementary Table). These results indicated that these viruses can present in three-toed jerboas and have the potential for intra-species transmission.

The HVs, designated RtDs-HV-1/IM2014, RtDs-HV-2/IM2014, and RtDs-HV/IM2015, were closely related to known *Murinae*-borne HTNV with 93% aa (89% nucleotide (nt)) identity for the L segment and 98% aa (89% nt) identity for the M segment. The partial sequences of the L and M segments of these three viruses shared 98–99% nt identities with one another. We propose the name Jerboa hantavirus (JEHV) for these new HTNV strains. To clarify the evolutionary relationships between JEHV and other HVs, phylogenetic analyses based on the partially deduced L and M proteins were conducted using MEGA6 (Fig. 1a, Supplementary Figure 1)^14^. The clade of JEHV in both phylogenetic trees revealed that these viruses could be classified into phylogroup III and clustered with *Murinae-*borne HTNV.

**Fig. 1:**
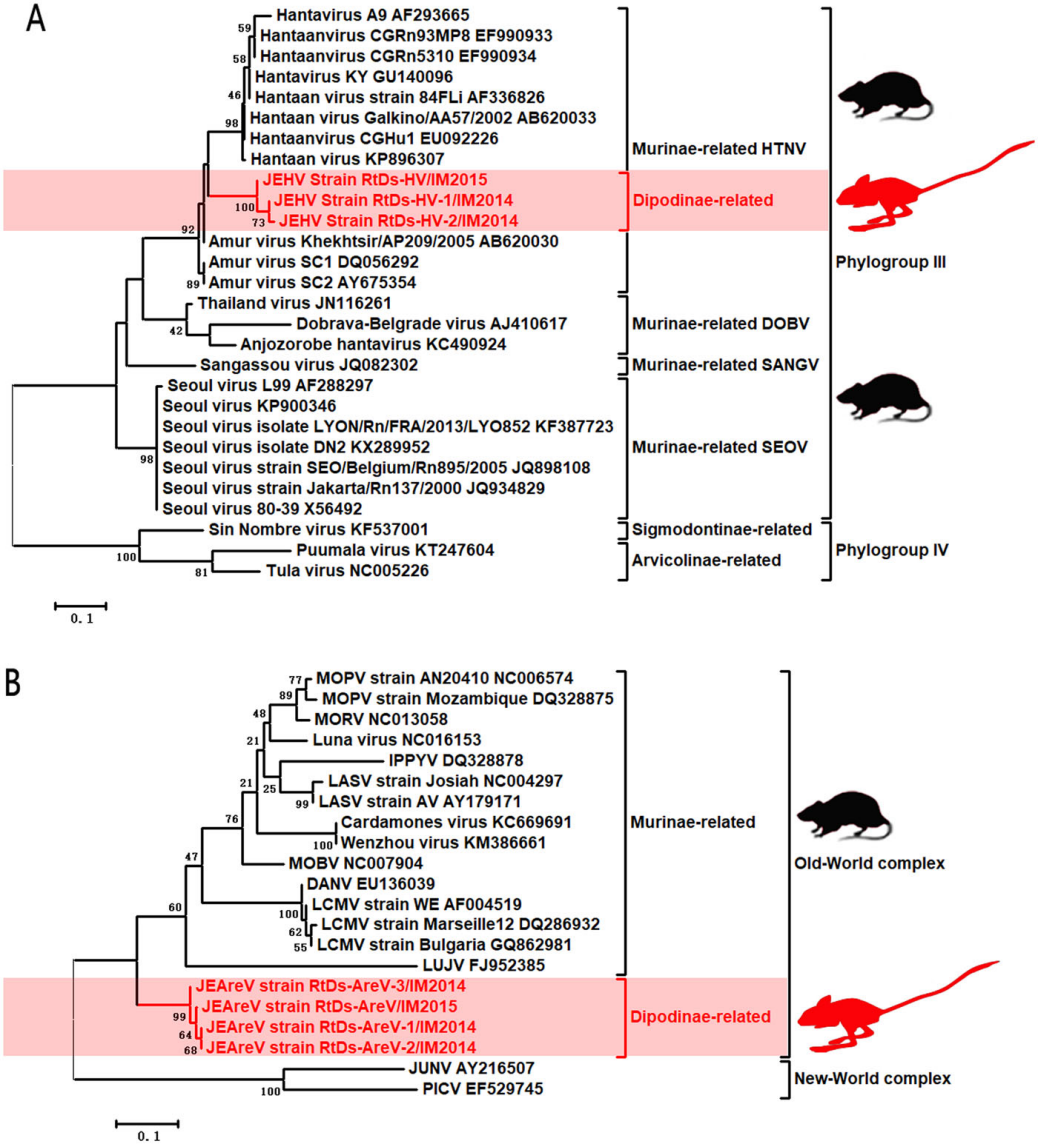
**a** Phylogenetic tree based on the deduced proteins of partial L segment (125 aa) of rodent-borne HVs. **b** Phylogenetic tree based on the partial L proteins (114 aa) of rodent-borne Old-World AreVs. MEGA6.0 was used to align deduced aa sequences using the MUSCLE package and default parameters. The best substitution model was then evaluated by the model selection package. Finally, we constructed a maximum-likelihood method using an appropriate model to process the phylogenetic analyses with 1000 bootstrap replicates. The viruses found in this study are labeled in red font. Scale bars indicate nucleotide substitutions per site

The AreVs, designated RtDs-AreV-1/IM2014, RtDs-AreV-2/IM2014, RtDs-AreV-3/IM2014, and RtDs-AreV/IM2015, were distinct from all known Old-World AreVs (<44% aa identity for the L protein, <63% aa identity for the glycoprotein, and <62% aa identity for the N protein). These four viruses shared 97–99% nt identities with one another, indicating that they belong to the same AreV species. In accordance with the species demarcation criteria of AreVs (https://talk.ictvonline.org/ictv-reports/ictv_9th_report/negative-sense-rna-viruses-2011/w/negrna_viruses/203/arenaviridae), we propose the name Jerboa arenavirus (JEAreV) for this new AreV species. Phylogenetic analyses based on the deduced L and G proteins were conducted using MEGA6 (Fig. 1b, Supplementary Figure 2). The two phylogenetic trees revealed that JEAreV could be assigned to the Old-World complex (69% bootstrap support). However, the location of the branches containing these viruses in the two phylogenetic trees indicated that this viral species appeared to have evolved separately and that it is distinct from all other *Murinae-*borne Old-World AreVs.

The present study shows that a *Dipodinae* member, the three-toed jerboa, can carry HTNVs and novel Old-World AreVs. Because JEHV and JEAreV were repeatedly detected in samples from three-toed jerboas but were not detected in samples from long-eared jerboas and five-toed jerboas, and no other rodent species were found from the same environment, we can infer that the three-toed jerboa might act as the natural reservoir of these viruses rather than as an intermediate host. This finding and its context indicate that HTNV and Old-World AreV are able to infect more mammalian hosts than previously thought and that *Murinae* species are not their only hosts. Considering that HV and AreV are among the most dangerous rodent viruses known to infect humans, the three-toed jerboa, a distinctive rodent species that lives in deserts and sandy ground and acts as a natural host of HV and AreV, deserves more attention in the prevention of hemorrhagic fever and related diseases transmitted by rodents.

## Electronic supplementary material

SUPPLEMENTAL MATERIALS(DOCX 124 kb)
