# Supplementary material for: Detection of Hantaviruses and Arenaviruzses in three-toed jerboas from the Inner Mongolia Autonomous Region, China
Source: Emerg Microbes Infect. 2018 Mar 21;7:35. doi: 10.1038/s41426-018-0036-y (PMC5861045; doi:10.1038/s41426-018-0036-y)

**Supplementary Table.** Prevalence of HVs and AreVs in rodents by species.

| Collection date | Species | HVs | AreVs |
| --- | --- | --- | --- |
| 2014.5 | Three-toed Jerboa (*D. sagitta*) | 2/26 | 3/26 |
|  | Long-eared Jerboa (*E. naso*) | 0/5 | 0/5 |
|  | Five-toed Jerboa (*A. sibirica*) | 0/28 | 0/28 |
| 2015.8 | Three-toed Jerboa (*D. sagitta*) | 1/24 | 1/24 |
|  | Long-eared Jerboa (*E. naso*) | 0/7 | 0/7 |
|  | Five-toed Jerboa (*A. sibirica*) | 0/15 | 0/15 |

**
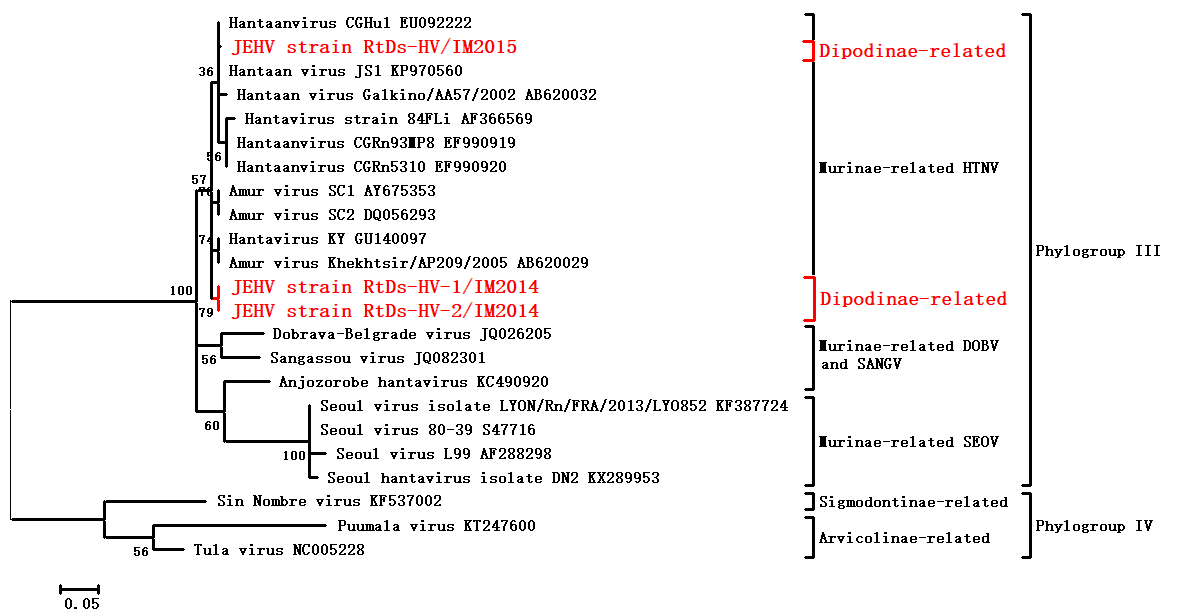
Supplementary Figure 1. Phylogenetic tree based on the deduced aa sequences of partial M segments (316nt) of rodent-borne HVs.**

**Supplementary Figure 2. Phylogenetic tree based on the complete G proteins of rodent-borne Old-World AreVs.**
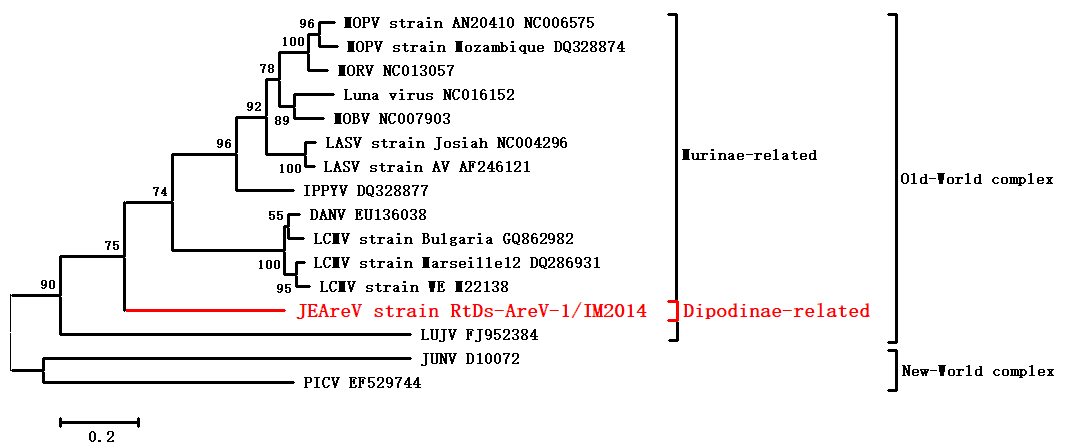

Supplement: Supplementary file 1 — SUPPLEMENTAL MATERIALS(DOCX 124 kb) [file 41426_2018_36_MOESM1_ESM.docx]
